# Critical Mutation Rate Has an Exponential Dependence on Population Size in Haploid and Diploid Populations

**DOI:** 10.1371/journal.pone.0083438

**Published:** 2013-12-27

**Authors:** Elizabeth Aston, Alastair Channon, Charles Day, Christopher G. Knight

**Affiliations:** 1 Research Institute for the Environment, Physical Sciences and Applied Mathematics, Keele University, Keele, Staffordshire, United Kingdom; 2 Faculty of Life Sciences, The University of Manchester, Manchester, Greater Manchester, United Kingdom; University of California Santa Barbara, United States of America

## Abstract

Understanding the effect of population size on the key parameters of evolution is particularly important for populations nearing extinction. There are evolutionary pressures to evolve sequences that are both fit and robust. At high mutation rates, individuals with greater mutational robustness can outcompete those with higher fitness. This is survival-of-the-flattest, and has been observed in digital organisms, theoretically, in simulated RNA evolution, and in RNA viruses. We introduce an algorithmic method capable of determining the relationship between population size, the critical mutation rate at which individuals with greater robustness to mutation are favoured over individuals with greater fitness, and the error threshold. Verification for this method is provided against analytical models for the error threshold. We show that the critical mutation rate for increasing haploid population sizes can be approximated by an exponential function, with much lower mutation rates tolerated by small populations. This is in contrast to previous studies which identified that critical mutation rate was independent of population size. The algorithm is extended to diploid populations in a system modelled on the biological process of meiosis. The results confirm that the relationship remains exponential, but show that both the critical mutation rate and error threshold are lower for diploids, rather than higher as might have been expected. Analyzing the transition from critical mutation rate to error threshold provides an improved definition of critical mutation rate. Natural populations with their numbers in decline can be expected to lose genetic material in line with the exponential model, accelerating and potentially irreversibly advancing their decline, and this could potentially affect extinction, recovery and population management strategy. The effect of population size is particularly strong in small populations with 100 individuals or less; the exponential model has significant potential in aiding population management to prevent local (and global) extinction events.

## Introduction

Small populations frequently exist in nature. Some animal species can exist in populations of only hundreds, while those nearing extinction may be found in populations of only a few individuals. The latter case is of particular concern. Understanding the effect of population size on the critical parameters of evolution (mutation, recombination, selection, and genetic drift) is essential in making accurate predictions regarding the likely fate of a small population if left to persist in its current environment. For example, inbreeding resulting from genetic drift in small populations can depress population fitness and increase the risk of extinction [1]. Environmental change is rapid, therefore populations need to evolve at a sufficient rate to prevent further population decline and enable evolutionary rescue [2]. Population decline can lead to loss of fit genetic material that may be difficult to recover in very small populations due to mutational meltdown [3].

Evolutionary systems are persistently under pressure to evolve sequences that are both fit and robust [4]–[6], where robustness is defined in terms of the average effect of a specified perturbation (such as a mutation) on the fitness of a specified genotype [7]. The greater the robustness, the smaller the change in fitness. The majority of mutations have a negative effect on fitness [8]; greater robustness to mutation can provide protection against loss of fitness and so can protect against the effects of such deleterious mutations. In addition, smaller populations are more susceptible to loss of fitness through genetic drift [9], [10].

The concept of a fitness landscape was introduced in [11] and later combined with the notion of sequence space in [12]. Each sequence in sequence space has a fitness value, which represents its relative replication capacity [13]. The fittest sequences in the landscape are the ‘peaks’, while the lower fitness sequences occupy the ‘valleys’. Sequence space is explored through evolution by mutation, recombination and selection (and so genetic drift) in accordance with the fitness landscape. Mutation introduces variation, while selection reduces it by removing low fitness sequences. The balance between these two forces is referred to as the mutation-selection balance [14], [15]. When there is mutation-selection balance, the population will tend to cluster around the fitness peaks and form a quasispecies, where a quasispecies consists of a distribution of genotypically closely related replicative units, centred around the copy corresponding to the phenotype of maximum selective value (the peak) [12], [15], [16].

Mutation introduces change at the sequence level. The greater the number of changes, the greater the chance of a beneficial mutation occurring. However, as the likelihood of detrimental mutations will also increase, changes occurring too frequently can lead to an inability of natural selection to maintain the population’s genetic makeup. In a landscape with a single fitness peak, a population is able to maintain its position surrounding the top of the peak so long as the mutation rate does not exceed a particular rate known as the error threshold. Above this threshold, there is an error catastrophe and the population delocalizes across sequence space [13], [15]–[20]. Note that this does not necessarily equate to an extinction threshold [21]. An error catastrophe is an evolutionary shift in genotype space, while extinction refers to the reduction of individuals in the population. A population that shifts to the lower fitness areas of the landscape is less well adapted to its current environment.

The concept of the error threshold was introduced in [22] and later in [23] based on the quasispecies equation:
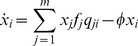
(1)


Here, 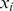 is the frequency of genotype number 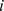, where 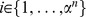, 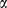 is the alphabet size, 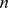 is the length of sequences, 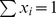, 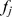 is fitness (selection), 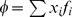 is the average fitness, and 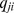 is a transition probability (mutation). The derivative in time is denoted 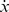, and there are 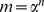 genetic sequences.

Selection and mutation provide two forces (or pressures) on the population, and they can be combined into one matrix 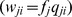 (see [16], p. 35). Selection draws the population closer to the highest fitness, while mutation is usually assumed to have a deleterious effect due to which the population drifts away from the highest fitness. Generally, the population converges to a stable (equilibrium) state that is defined by an eigenvector of the mutation-selection matrix 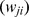. This eigenvector corresponds to the largest eigenvalue of 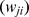, which is the average fitness 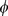 [16]. The error threshold is dependent on the existence of a mutation-selection balance when the effect of mutation does not exceed that of the selection pressure; it is the maximal mutation rate that allows a population to stay clustered around the fitness peak. Note that Equation 1 is a model for infinite populations. So, strictly speaking, the error threshold does not exist when 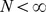. However, Equation 1 can be used as an approximation for finite population dynamics [24]. The dynamics of finite populations have been studied for a long time in single-peak landscapes [25], [26]. They have also been studied using the Moran process [16], [27]. The discrete-time formulation of the quasispecies equation has been used to describe mutation-selection dynamics [28]–[30].

An error catastrophe can delay or prevent extinction by shifting the population to more robust genotypes [21]. In addition to the error threshold, in landscapes where there is more than one peak, there may also be one or more critical mutation rates at which the population loses its ability to remain on fitter peaks, but retains its ability to remain on flatter peaks of lower fitness [9], [17], [29], [31]. Above such critical mutation rates, individuals with greater robustness to mutation are able to survive while fitter, less robust individuals may not. This represents a phase transition from survival-of-the-fittest to survival-of-the-flattest [6], [9], [15], [20], [29], [31], [32]. At lower mutation rates, selection favours individuals that reside at peaks with higher fitness, due to the rarity of mutations that push individuals off the peaks [5]. However, at higher mutation rates there will be an increase in the frequency of mutations which push individuals off the peaks; selection favours individuals located in flatter regions of the fitness landscape as individuals here are less likely to experience large reductions in fitness compared with those that may be initially fitter but reside in parts of the landscape with steeper peaks. Individuals that are part of a neutral network [33]–[35], in that they are connected in sequence space to other individuals with equivalent fitness, are said to be more mutationally robust than individuals that are not [15], [36]–[38]. The critical mutation rate has been defined as the midpoint between the highest mutation rate at which there is survival-of-the-fittest, and the lowest mutation rate at which there is survival-of-the-flattest [9], [31].

Survival-of-the-flattest has been observed in digital organisms [31], [32], theoretically [32], [37], in simulated RNA evolution [38], and in RNA viruses [6]. Evolution of mutational robustness has also been observed in simulated RNA evolution [39], in an artificial evolution model with digital organisms [40], and in laboratory protein evolution experiments [41]. Both [39] and [41] place an emphasis on the degree of polymorphism in the population, suggesting that highly polymorphic populations are more likely to spread across many nodes of a neutral network (each corresponding to a genotype), concentrating at highly connected parts; individuals at highly connected nodes have greater robustness to mutation, which they pass on to the next generation. Flat landscapes have been referred to as redundant, and steeper landscapes as antiredundant. It has been suggested that both in theory and in individual-based stochastic simulations, redundancy increases the mean fitness in small populations as it masks mutations that arise due to mutational drift [42]. However, large populations are less affected by drift, and so are more able to occupy high-fitness peaks in sharp landscapes.

Both [38] and [9] found “that population size played only a minor role in determining the position of the critical mutation rate” [29], within the context of their experiments. Population sizes as low as 250 were used, and the conclusion made “that the critical mutation rate was independent of population size” despite the fact that there did appear to be some correlation for certain cases [9]. They did not consider smaller populations, such as those that may exist for species nearing extinction or living in localized groups. Both [23] and [43] considered the effect of random genetic drift in finite populations (in haploids and diploids respectively), and observed that there is a shift of error thresholds to lower values which is more pronounced the smaller the population. Error thresholds were also shown to increase for increasing population size using a genetic algorithm with both single-peak and correlated landscapes [44]. Based on these results for error thresholds, we consider the need for further investigation of the critical mutation rate at smaller population sizes than those previously studied, and pose first the following hypothesis:

### Hypothesis 1

Critical mutation rate has a dependence on population size in haploid populations.

It should be noted that the parts of this paper associated solely with hypothesis 1 were presented at the European Conference on Artificial Life (ECAL 2011) [45]. All mammals have two copies of their genome; they are diploid as opposed to haploid [46]. In diploid organisms, one copy of the genome is inherited from the mother, while the other is inherited from the father. Each individual will therefore have two copies of each gene, each of which may be of a different form (a different allele). Different alleles have different degrees of dominance; an individual with two different alleles will display the phenotype of the dominant allele. In the majority of cases, mutant alleles are recessive while the non-mutant wild-type alleles are dominant [47].

The error threshold has been studied in a single-peak fitness landscape with a diploid population [48]. The quasispecies model was used, in which a molecule is represented as a string of 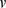 digits, each of which is allowed to be one of 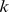 different values representing the different types of monomer used to make the molecule. The 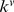 different strings can be considered as different alleles of a gene that determines the fitness of a haploid individual; this closely follows the classical one-locus, multiple-allele model of population genetics [10]. A diploid analogue of the single-peak fitness landscape was used, in accordance with the quasispecies model which was generalized by [43] to consider diploid individuals. There is a dominance parameter 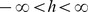, where the master allele is completely dominant for 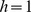 and completely recessive for 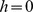. If 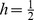, there is no dominance. In addition to this, 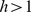 models the case where there is heterozygote advantage, while 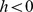 models heterozygote disadvantage. It was observed that, for 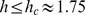, there are two distinct regimes: the quasispecies regime in which there is a single master allele around which most of the population is situated in sequence space, and the uniform regime where the 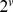 alleles appear in the same proportion. They define the error threshold as being the error rate at which the transition between these two regimes occurs, with 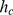 representing a critical value beyond which the two regimes can no longer be distinguished. Beyond the error threshold the system undergoes an error catastrophe, something which was found to be postponed or even avoided in the case of a dominant allele 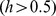. Based on the presence of an error threshold for a diploid population as described by [48], it is expected that the relationship between population size and critical mutation rate observed for a haploid population should be conserved to some degree when moving from haploidy to diploidy:

### Hypothesis 2

Critical mutation rate has a dependence on population size in diploid populations.

As diploid individuals have two copies of each sequence, this may confer a greater degree of robustness as any deleterious mutation will be potentially cancelled out; the second sequence has the potential to provide a back-up copy. This increased robustness may allow diploids to withstand higher mutation rates, and therefore have higher critical mutation rates and error thresholds than haploids.

We present five contributions. First, an algorithmic method, operating at the level of the individual, which does not rely on the precise details of the underlying fitness landscape and is therefore capable of providing widely applicable results. Second, verification of the method against analytical models for the error threshold, providing confidence in our subsequent results. Third, the discovery of an exponential relationship between the critical mutation rate and population size in haploid populations (Hypothesis 1). Fourth, the result that this is conserved when moving from haploidy to diploidy (Hypothesis 2) but that the critical mutation rate and error threshold are both unexpectedly lower in the latter case. Fifth, an analysis of the transition from critical mutation rate to error threshold (survival-of-the-fittest to survival-of-the-flattest) which provides for an improvement on previous definitions of the critical mutation rate. These contributions provide the key insight that the critical mutation rate, at which individuals with greater robustness to mutation are favoured over individuals with greater fitness, has an exponential dependence on population size in both haploid and diploid populations, the latter in a system modelled on the biological process of meiosis. This is in contrast to previous studies which identified that critical mutation rate was independent of population size. Our results show the effect of population size to be particularly strong in small populations with 100 individuals or less.

## Methods

### Haploid Method

**Figure 1:**
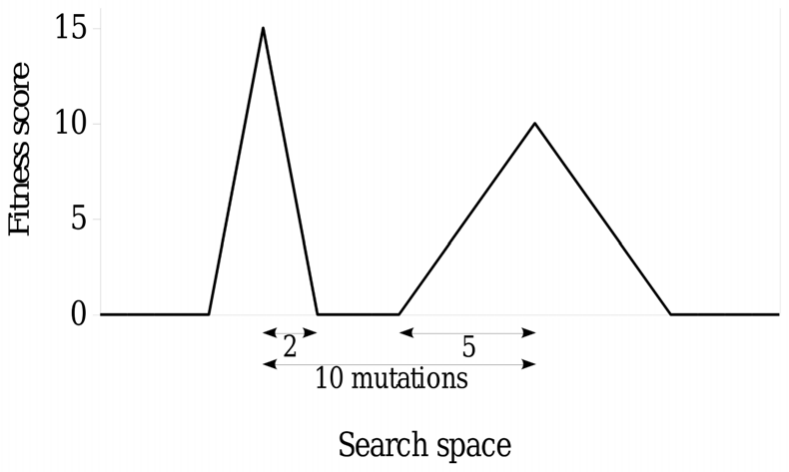
Two-peak fitness landscape. There is one narrow peak of high fitness (peak 0), and one broader, flatter peak of lower fitness (peak 1).

An individual sequence consists of a string of characters drawn from an alphabet of size 4 (which can be thought of as, for example, A/C/G/T or 0/1/2/3) with a fixed length of 30. In each step of the algorithm, three individual sequences are selected at random from the population. Two of the three selected individuals are chosen as parents in a crossover which replaces the third individual with the resulting child. The child is then subject to one round of point mutation (to a *different* base) at a given per-base mutation rate. The individual to be replaced is determined each time based on the fitnesses of the three selected individuals: there is an equally small chance of either of the two fittest of the three being replaced (25%), and a larger chance of replacing the least fit (50%) The 25∶25∶50 ratio ensures that any individual can be chosen, so allowing a population to lose its fittest peak. This use of tournament selection ensures that selection is independent of the precise shape of the landscape. This process continues until each individual in the population has been chosen exactly once (or there are less than three remaining to select); this represents one generation. The fitness of each individual sequence is evaluated based on a two-peak fitness landscape with one narrow peak of high fitness (peak 0), and a broader, flatter peak with lower fitness (peak 1) (Figure 1). Peak 0 has a maximum fitness score of 15 and a radius of 2, where radius refers to the Hamming distance from top-of-peak to zero fitness score. Peak 1 has a maximum fitness score of 10 and a radius of 5, with its top chosen as an arbitrary point (fixed throughout evolution) with a Hamming distance of 10 from the top of peak 0. This is done by setting the sequence at the top of peak 0 to be a string of 0 s, while the sequence at the top of peak 1 is set as a string of 0 s with 10 of those 0 s randomly changed to either a 1,2 or 3. Individuals are allowed to move on the slopes, or in between the peaks. This is a simple landscape in which survival-of-the-flattest can occur, with generality due to the use of tournament selection. The effect of mutation on fitness is smaller within peak 1 than within peak 0; individuals located on peak 1 will have higher mutational robustness compared with those located on peak 0.

Following the experimental procedure designed by [31] (and used by [9]) we initialized half of the population to peak 0 and half to peak 1 to avoid any initial bias towards either peak. The simulation was run for 10,000 generations, and the first generation at which there were no individuals on peak 0 was recorded. If peak loss did not occur within the 10,000 generations, a value of −1 was recorded in place of the generation number. Similarly, the number of generations it took to lose peak 1 was also recorded. A range of per-base mutation rates was tested for a range of population sizes, with the simulation being repeated and run 2000 times for each combination. It should be noted that the population size is fixed for the duration of each run. The mutation rate by which 95% of the runs had lost each peak was recorded as the critical mutation rate, where a peak was considered to have not ever been lost only if there were individuals remaining on it at the end of the 10,000 generations.

### The Moran Process

Evolution in finite populations can be described using the Moran process [16]. The Moran process is a simple birth-death process in which each time step involves choosing at random one individual for reproduction and one individual for death. Death occurs by replacing the latter individual with the child of the former individual. There are no restrictions to ensure each individual is chosen any number of times. Nowak and Schuster use a system based on the Moran process [23], in which they group individuals into error classes (where individuals are in the same error class if they are the same Hamming distance away from the master or target sequence). They take into account the number of individuals in each error class to calculate the transition probabilities of the birth and death process. The haploid method described above is a variation on this; population mixing is done through crossover of two reproducing sequences to replace a third sequence marked for death, with every individual being chosen once and only once to provide a defined generation. Use of crossover to introduce mixing is a more biologically realistic process. In addition, while Nowak and Schuster’s method considers frequency at the level of the population, the haploid method described above operates at the level of the individual sequence.

### Diploid Method

The genetic algorithm for a diploid population was modelled on the biological process of meiosis. Meiosis is a type of cell division which produces haploid cells. DNA is made up of two complimentary sequences (double-stranded), and condenses during cell division to form structures known as chromosomes [49]. Diploid organisms have two copies of each chromosome. For example, humans have 46 chromosomes in 23 pairs. One of each pair comes from the father, the other from the mother. The pairs are known as homologues. Each chromosome is replicated (during which process there is a chance of mutation), and subsequently becomes a complex made up of two identical sister chromatids which form an X-shaped structure (bivalent). Homologous chromosomes join together to form a tetrad. This means, for example, that the maternal copy of chromosome 1 will pair up with the paternal copy of chromosome 1. It is called a tetrad as it is made up of four chromatids (the original maternal and paternal chromosomes and their duplicates). Crossover occurs within the tetrads. The pairs are pulled apart to opposite ends of the cell. Each end of the cell will subsequently have one copy of the chromosome. The cell splits to create two cells, each with the correct number of chromosomes (one copy of each). Each cell will contain a mix of paternal and maternal DNA due to crossover. In each of the two cells, the chromosomes are split into their constituent chromatids. The chromatids are pulled to opposite ends, and the cells divide. The result is four cells, each containing one chromatid (now referred to as a chromosome). The resulting four cells are haploid as they contain only one copy of each chromosome, and are known as gametes. The joining of a gamete from a mother with that from a father will produce a diploid child.

In the genetic algorithm, each genetic sequence is represented as a string of 30 characters. DNA is double-stranded, but as one strand is just a compliment of the other, it can be represented as a single-strand string in the simulation. Consistent with the haploid system, each character in the sequence is one of four possibilities. A diploid individual consists of two sequences, one inherited paternally and one inherited maternally. There are no distinct sexes in the simulation; the terms *maternal* and *paternal* are used merely to differentiate between the two parent individuals. At the start of the algorithm, each individual is initialized so that both of its constituent sequences are identical (homozygous). In each step of the algorithm, three individuals are selected from the population at random. Two of the selected individuals are chosen to be parents, while the third will be replaced by their child after reproduction. Selection is carried out based on the fitness of the three individuals. There is an equally small chance of either of the two fittest individuals being chosen to be replaced (25%) and a higher chance of the individual with the lowest fitness being replaced (50%). After selection, crossover occurs within each parent individual between the maternal and paternal sequences. A locus is randomly selected to be the crossover point. The maternal sequence is copied up to this locus, and the paternal sequence after. This produces a single-sequence gamete from each parent, the bases of which are then mutated (each to a different base) according to a per-base mutation rate. One of the gametes is randomly designated the paternal sequence for the child, while the other becomes the maternal. The resulting diploid child becomes part of the population. This process continues until each individual in the population has been chosen exactly once (or there are less than three remaining to select); this represents one generation and ensures that there is no chance of any individual avoiding being chosen and so remaining static in the landscape. The fitness of each individual sequence is evaluated based on the two-peak fitness landscape (Figure 1) and the experimental procedure is that used in the haploid system.

### Fitness Calculation

The key difference between the haploid experiment described above (and in [45]) and the diploid experiment is the introduction of diploidy. In the haploid case, the fitness of each individual is calculated based on the Hamming distance of an individual sequence from the top of each peak. The fitness of the individual in terms of peak 0 is equal to 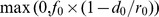, where 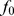 is the fitness score of the target at the top of peak 0, 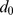 is the Hamming distance of the individual from this target, and 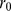 is the Hamming distance between the target and the point at which the peak has a fitness score of 0 (see Figure 1). The fitness of the individual is also calculated in terms of peak 1. The higher of the two fitness values is designated to be the overall individual fitness score. However, a diploid individual consists of two sequences and has a fitness score for each. To obtain an overall individual fitness score, 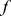, we introduce a dominance parameter which we denote 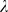:
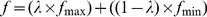
(2)


The fitness score for each of the constituent sequences is compared. The sequence with the higher of the two fitnesses has its fitness score designated 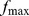, while 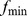 is the fitness score for the sequence with the lower fitness. If both sequences have the same fitness, 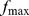 and 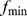 will have equal value. When 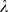 is set equal to 1, the overall individual fitness is equal to the maximum of the two fitness scores. When 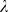 is set equal to 0, the overall individual fitness will be equal to the minimum of the two fitness scores. The experiment was run with 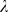 set at a range of values, 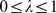.

## Results

### Observed Error Thresholds are Consistent with Analytical Models

We developed an algorithmic method that simulates evolution of a haploid population on a two-peak landscape (see the Methods section below and Figure 1). Using this, we measure both the critical mutation rate and the error threshold for a range of population sizes. Nowak and Schuster [23] use a system based on the Moran process and present an analytical expression for the population size dependence of the error threshold (Equation 3), where 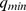 is the error threshold, 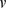 is sequence length, 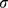 is the selection strength or superiority parameter of the master (fittest) sequence, 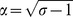, and 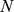 is population size:
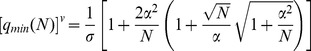
(3)


Ochoa et al. [50], [51] derived a reformulation of the Nowak and Schuster analytical expression (Equation 4), in which they make explicit the reduction in the error threshold when moving from infinite populations to those of size 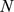 (see [50] section 3 for the detailed derivation). Here 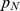 is the error rate:
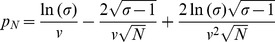
(4)


**Figure 2:**
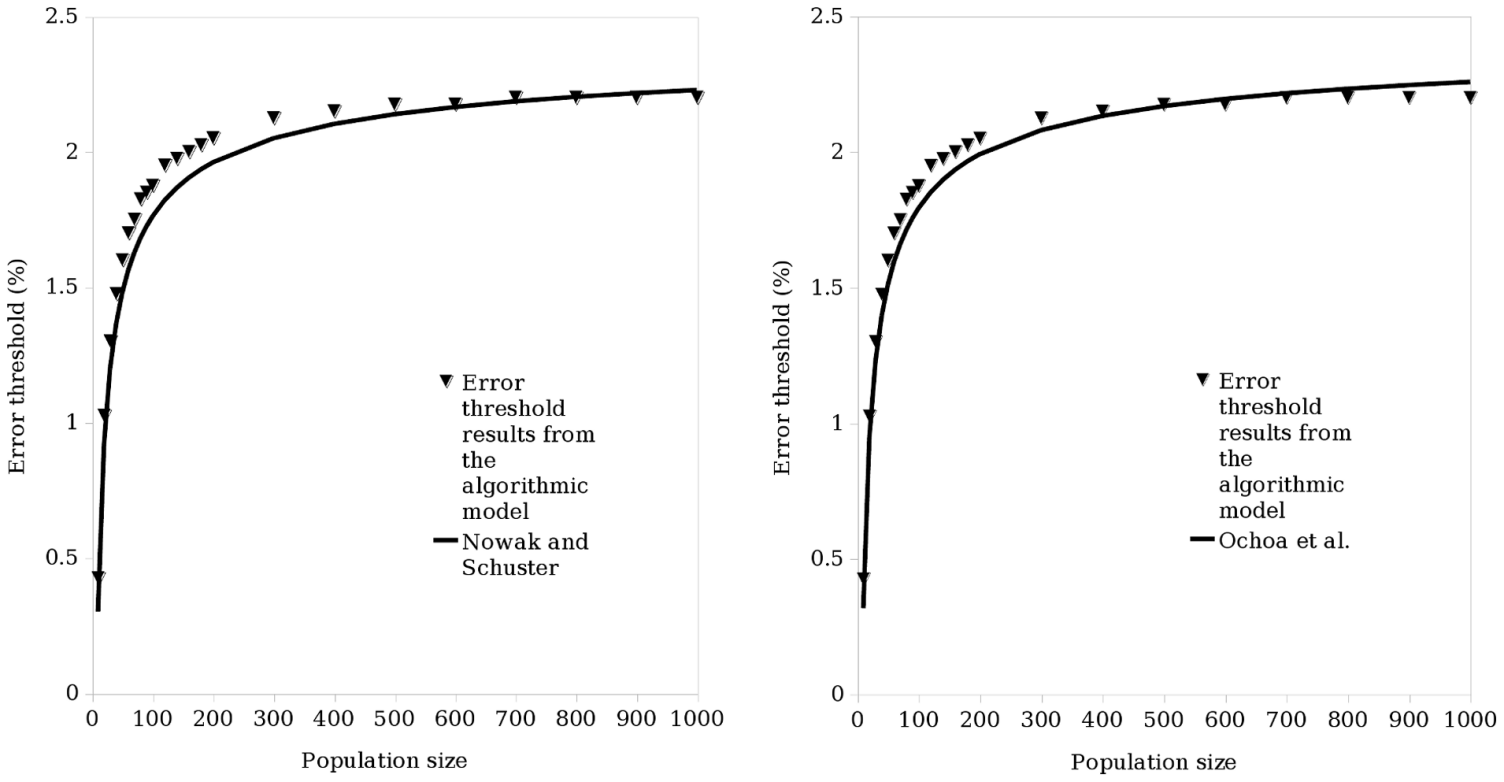
Verification of the method against analytical models for the error theshold. Nowak and Schuster [23] present an analytical expression for the population size dependence of the error threshold (Equation 3). Ochoa et al. [50], [51] include a reformulation of the Nowak and Schuster analytical expression (Equation 4), in which they make explicit the reduction in the critical mutation rate when moving from infinite populations to those of size 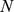 (see [50] section 3 for the detailed derivation). The observed consistency between our results and the analytical models provides verification for our results and the algorithmic method as a whole. It should be noted that the 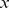 axis represents the mutation rate by which 95% of runs have lost the lower, flatter peak (peak 1).

Figure 2 shows the error thresholds from our algorithmic method alongside those from Equations 2 and 3 using a 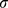 value of 2.1. It should be noted that 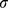 is the superiority parameter which would normally be calculated as the ratio of the two fitness peaks. However, as fitness in our algorithmic method is represented as a score as opposed to being a direct measure of reproductive rate, and selection is determined only by fitness score rank, independent of the magnitude of fitness score difference (such that any strictly monotonic transformation of fitness score would produce the same results), we show here the curves with the 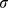 value that best fits the complete range of our results. It has been confirmed that changing the original algorithmic method to include peak heights with a ratio of 2.1 produces a comparable match. The observed consistency with the analytical models provides verification for our algorithmic method, and therefore confidence in our subsequent results.

### Critical Mutation Rate has an Exponential Dependence on Population Size in Haploid Populations

**Figure 3:**
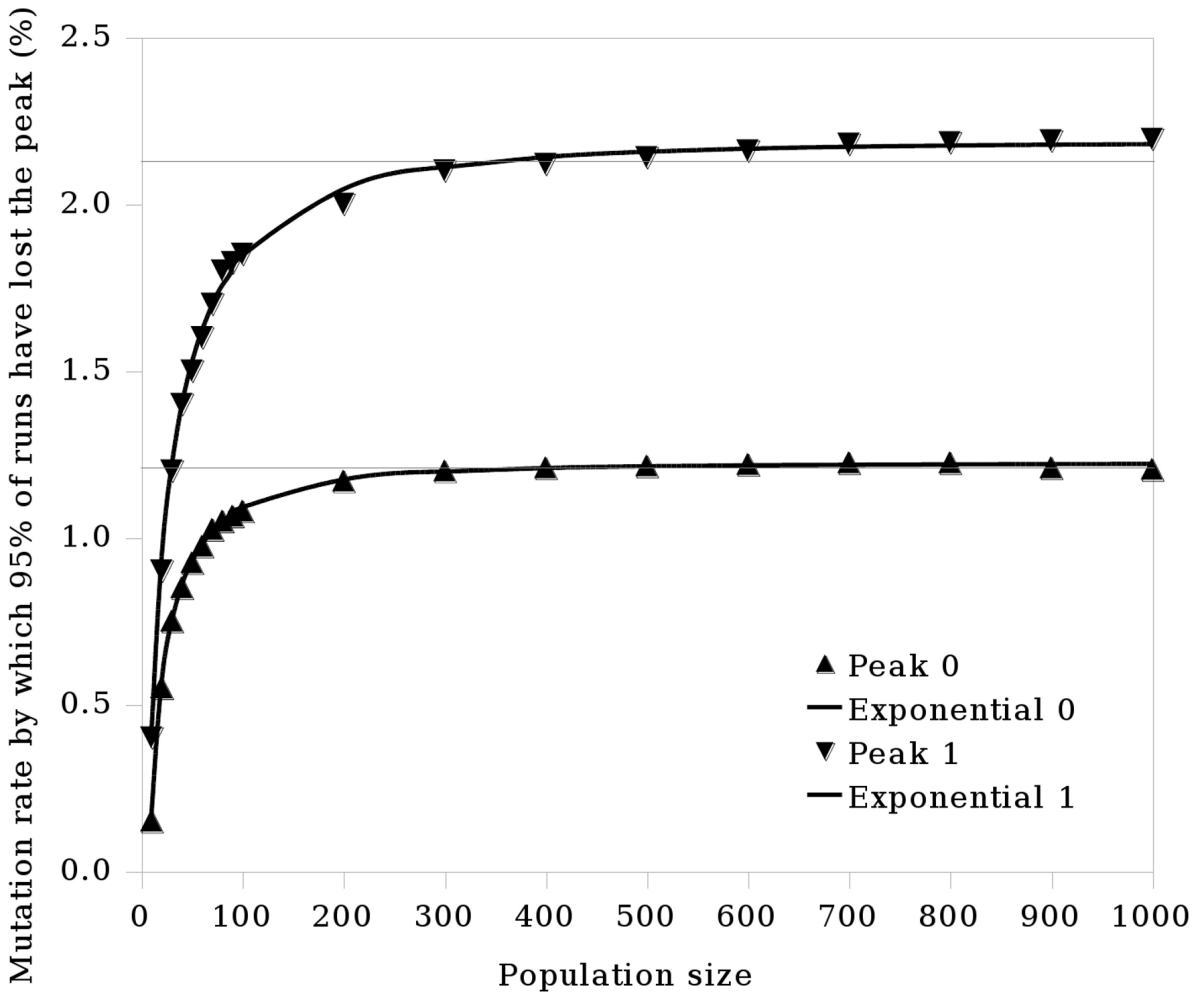
The results of the simulation can be approximated by an exponential function. This applies to both peak 0 (high, narrow peak) and peak 1 (lower, flatter peak). 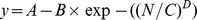 (with 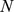 being population size). The parameters (and their standard error in brackets) obtained by curve-fitting using a least squares method were, for the high, narrow peak (peak 0): 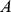 = 1.221% (0.0033%), 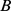 = 7.001% (1.4390%), 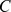 = 1.440 (0.1701), 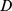 = 0.3250 (0.02739), and for the lower, flatter peak (peak 1): 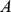 = 2.184% (0.0122%), 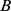 = 5.438% (1.0466%), 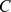 = 7.721 (0.2734), 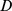 = 0.3978 (0.0476).

Using a population of haploid individuals and a genetic algorithm with a simple two-peak fitness landscape (Figure 1), we find that the mutation rates at which the high, narrow peak and the lower, flatter peak are lost (the survival-of-the-fittest and survival-of-the-flattest regimes ending at the critical mutation rate and error threshold respectively) for increasing population sizes can be approximated by an exponential function (Figure 3).

**Figure 4:**
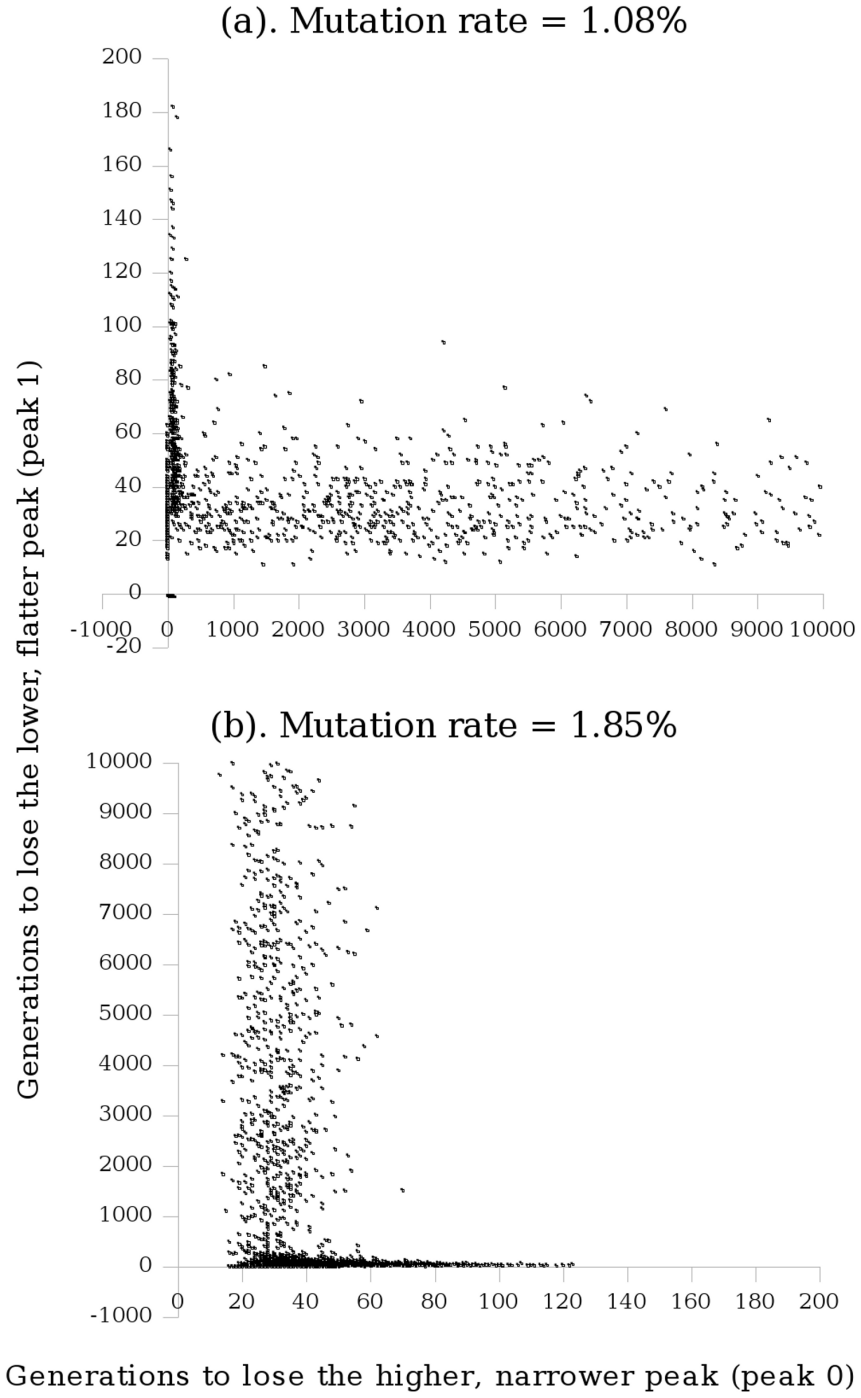
Transition from survival-of-the-fittest to survival-of-the-flattest and subsequently to the error catastrophe. Each point represents the number of generations it took to lose the high, narrow peak (peak 0) and the number to lose the lower, flatter peak (peak 1), in a single run of the GA for population size 100, sequence length 30. Where a peak was not lost within 10,000 generations, a value of −1 was assigned for that particular run of the genetic algorithm: all points on the negative side of either axis should be taken to have a value greater than 10,000.

As opposed to there being instantaneous transitions from survival-of-the-fittest to survival-of-the-flattest and to the error catastrophe at discrete mutation rates, there are gradual transitions in which there are shifts from the first to the second, and from the second to the third (Figure 4). The mutation rate at which 95% of the runs have lost the high, narrow peak (peak 0) within 10,000 generations marks a point at which the transition from survival-of-the-fittest to survival-of-the-flattest is essentially complete. This can be considered as a critical mutation rate. For a haploid population of 100 individuals of length 30, this is at a per-base mutation rate of approximately 1.08%. Figure 4(a) shows the number of generations taken to lose each peak at this mutation rate, for each of the 2,000 runs. Just 52% of these runs lost peak 1 within the duration of the simulation (compared to 95% for peak 0). Loss of peak 0 is then followed by one of two events: either peak 1 is lost relatively quickly (within 200 generations) or it is maintained for the duration of the simulation. The fate of the population after loss of peak 0 is therefore dependent on whether or not it is able to quickly converge on peak 1. Figure 4(a) shows (at this mutation rate) that when peak 0 is not lost early, the number of generations taken to lose peak 0 is distributed approximately evenly up to 10,000 generations.

The mutation rate corresponding to 95% of the runs having lost the lower, flatter peak (peak 1) within 10,000 generations marks a point at which the transition from survival-of-the-flattest to the error catastrophe is essentially complete. This can be considered as another critical mutation rate (or the error threshold). For a haploid population of 100 individuals of length 30, this is at a per-base mutation rate of approximately 1.85%. Figure 4(b) shows the number of generations taken to lose each peak at this mutation rate, for each of the 2,000 runs. It is an apparent reversal of Figure 4(a) but with 100% of the runs having lost peak 0 within 200 generations. The population has almost entirely lost the ability to localize to either peak.

### The Relationship between Critical Mutation Rate and Population Size is Conserved when Moving from Haploidy to Diploidy

**Figure 5:**
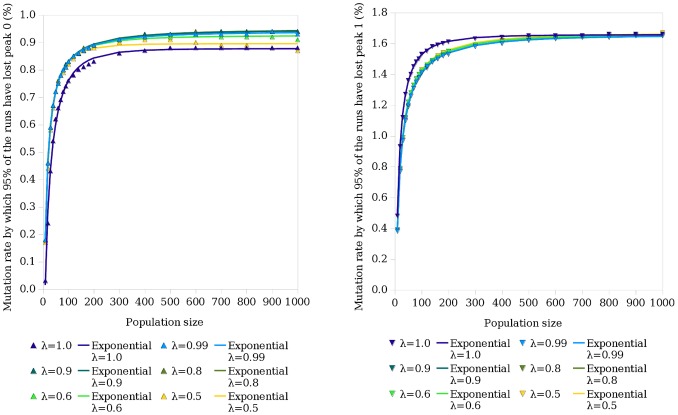
The relationship between population size and critical mutation rate is consistent across haploids and diploids. Here 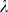 is the dominance parameter, as described in the section entitled Fitness Calculation. The simulation was run using the 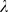 values listed. The points show the results obtained, which can be approximated by exponential functions as shown by the lines (obtained by curve-fitting using a least squares method). The left graph shows the curve obtained for the critical mutation rate and the right graph shows the error threshold, both for a diploid population. Refer to Figure 3 for the equivalent curves for a haploid population.

**Figure 6:**
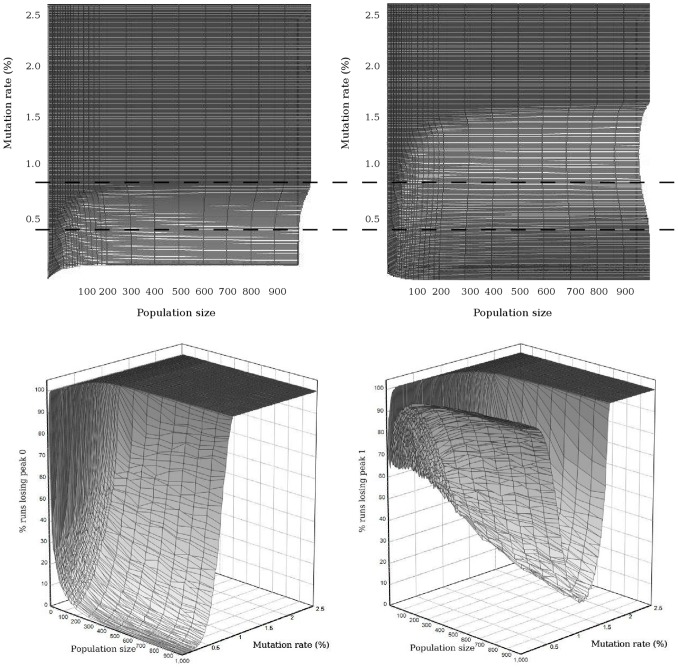
Percentage of runs losing the peaks at different mutation rates and population sizes. The results shown are for the diploid method with 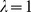, for peak 0 (a, left) and peak 1 (b, right). In the two lower projections the axis coming out of the page is the percentage of runs. The lower dashed line across these projections indicates, for population sizes of several hundred individuals, approximately where the percentage loss of peak 0 begins to rise steeply and that of peak 1 begins to fall steeply as mutation rate is increased: the transition from survival-of-the-fittest to survival-of-the-flattest. Likewise, the upper dashed line indicates approximately where the percentage loss of peak 0 has reached 100% and that of peak 1 has reached its minimum before rising back upward as mutation rate is increased further: the transition from survival-of-the-flattest to the error catastrophe.

Based on the observation that the error threshold has a dependence on haploid population size, and the observation by [43] that this relationship is not lost in diploid systems, a hypothesis was formed that the relationship will also hold for the critical mutation rate in haploid and diploid systems with a two-peak landscape. In a diploid system modelled on the process of meiosis in biology, each individual has two copies of the genetic sequence and recombination occurs within as opposed to between individuals. The resulting single-sequenced gamete then joins with another to form a child. The haploid and diploid methods of reproduction are fundamentally different; single-sequence versus two-sequence individuals, and between-individual recombination versus within-individual recombination means two populations reproducing using the two different systems will differ in their occupation of sequence space. The two copies of each sequence present in diploid individuals also gives them a redundancy not found in haploids. It was therefore expected that there would be some variation in the results when the experiments with a haploid system were reproduced using a diploid system. Consistent with this, the results using the haploid system also apply to a diploid population, but the diploid critical mutation rate and error threshold curves are lower than those for a haploid population (Figures 3, 5 and 6).

Transition between the states shown in Figure 4 is maintained when moving from haploidy to diploidy. Visualizing the relationship between population size, mutation rate and percentage of runs losing each peak shows the continuous transition from survival-of-the-fittest to survival-of-the-flattest (around the critical mutation rate) and subsequently to the error catastrophe (around the error threshold), and emphasizes the relationship between these transitions (Figure 6). For example, for population sizes of several hundred individuals, the lower dashed line across the lower projections in Figure 6 indicates approximately where the percentage loss of peak 0 begins to rise steeply and that of peak 1 begins to fall steeply as mutation rate is increased: the transition from survival-of-the-fittest to survival-of-the-flattest; and the upper dashed line indicates approximately where the percentage loss of peak 0 has reached 100% and that of peak 1 has reached its minimum before rising back upward as mutation rate is increased further: the transition from survival-of-the-flattest to the error catastrophe. In the upper projection (b) of Figure 6 it can be seen that for smaller population sizes (less than 50) the percentage of runs losing peak 1 does not fall below approximately 70%. This suggests 70% loss of peak 1 as a lower bound when considering error threshold. Below 50% loss of peak 0, individuals have transferred from peak 1 to peak 0, so 50% is a lower bound for considering critical mutation rate. The shapes of the population size to mutation rate mappings become increasingly consistent as we move above these lower bounds and 95% peak loss is a good choice for both critical mutation rate and error threshold.

## Discussion

In a fitness landscape, the fittest sequences are the ‘peaks’, while the lower fitness sequences occupy the ‘valleys’. Sequence space is explored through evolution by mutation, recombination, selection and genetic drift in accordance to the fitness landscape. Mutation introduces variation, while selection acts to increase the frequency of fitter sequences. The first contribution of this study is the development of an algorithmic method that operates at the level of the individual, in which selection is independent of the precise shape of the underlying landscape. The second contribution is the verification of this method using equations from analytical models (Equations 3 and 4) to produce comparable curves (Figure 2). Nowak and Schuster [23] present an analytical expression for the population size dependence of the error threshold using a system based on the Moran process (Equation 3). In Nowak and Schuster’s system there is no crossover; population mixing is instead achieved by calculating transition probabilities based on the number of individuals that are a certain Hamming distance away from the master sequence (see section ‘The Moran Process’). This is comparable to our algorithmic method which introduces mixing through the biologically realistic process of crossover. Ochoa et al. [50], [51] include a reformulation of the Nowak and Schuster analytical expression (Equation 4), in which they make explicit the reduction in the error threshold when moving from infinite populations to those of size 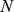 (see [50] section 3 for the detailed derivation). The observed consistency with the analytical error threshold models provides verification for our critical mutation rate results and our algorithmic method as a whole.

The third contribution of this work is to show that, for a haploid population and a two-peak landscape, the mutation rates at which the high, narrow peak and the lower, flatter peak are lost for increasing population sizes (of individuals of length 30) can be approximated by an exponential function. The null hypothesis 1 (that critical mutation rate has no dependence on population size in haploid populations) can therefore be rejected. The effect of population size is particularly noticeable in populations of 100 individuals or less. We also observe that the curve obtained for the critical mutation rate flattens out to a greater degree than the curve obtained for the error threshold. This can be seen by looking at the faint lines in Figure 3. It is also noticeable by the difference in the value of the 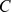 parameter defined in Figure 3’s caption, where 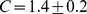 for the critical mutation rate and 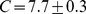 for the error threshold; the lower the value of 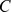, the flatter the curve. This explains why previous studies of larger populations have concluded that there is no relationship between the critical mutation rate and population size (e.g., [9]).

Using a genetic algorithm based on the biological process of meiosis, our fourth contribution is to demonstrate that the exponential relationship is conserved when moving from haploidy to diploidy, but that the critical mutation rate curves observed for a diploid system are lower than those observed for a haploid system (Figure 5). The null hypothesis 2 (that critical mutation rate has no dependence on population size in diploid populations) can therefore be rejected. It has been suggested that there is an interaction between mutation rates and mating strategies in nature [52]. Haploid systems use between-individual recombination while diploid systems use within-individual recombination. Recombination lowers the mutation rate at which the error threshold occurs [53]. Assortative, non-random mating, in which individuals of a similar phenotype mate more often than expected by chance, is able to overcome this shift toward lower error threshold magnitudes induced by recombination [52]. Conversely, dissortative mating, in which dissimilar individuals mate more often, reduces the magnitude of the error threshold. In the haploid system, the simulation starts with the population clustered at the two peaks. As the simulation is run, the population tends towards one of the peaks assuming the mutation rate does not exceed the error threshold. Recombination therefore tends to occur between sequences with similar fitnesses, and mating can be considered to be assortative. In our diploid system, the simulation starts with the population clustered at the two peaks, with individuals either completely at either peak, or with one sequence at one peak and one at the other. As fitness is calculated as a single value based on the fitness of an individual’s two constituent sequences (see section entitled Fitness Calculation), an individual can have, for example, a high fitness value but consist of two sequences in completely different parts of the fitness landscape. There is therefore a chance that the individuals selected to mate could have very different genetic make-ups; the degree of dissortative mating exceeds that of the haploid system. We suggest the difference in mating systems used by haploids and diploids as a potential reason for the difference in the curves shown in Figure 5; further work will be required to confirm this.

The fifth contribution of this work is the development and improvement of the definition of the critical mutation rate following analysis of our results. Previous studies have defined the critical mutation rate to be the midpoint between the highest mutation rate at which there is survival-of-the-fittest, and the lowest mutation rate at which there is survival-of-the-flattest [9], [31]. However, the results of our study clearly show that there is a transition from survival-of-the-fittest to survival-of-the-flattest and subsequently to the error catastrophe (Figure 4). Figure 4(a) shows that 95% of the runs had lost peak 0 within the duration of the simulation when the per-base mutation rate was 1.08%; just 52% of these runs lost the lower, flatter peak (peak 1). At this point, the transition from survival-of-the-fittest to survival-of-the-flattest is essentially complete. This can be considered as a critical mutation rate. Figure 4(b) shows that 95% of the runs had lost peak 1 within the duration of the simulation when the per-base mutation rate was 1.85%; 100% of these runs lost peak 0. This demonstrates that the transition from survival-of-the-flattest to the error catastrophe is essentially complete, with the population having almost entirely lost the ability to localize to either peak at this mutation rate. Figure 6 shows these transitions occurring in a diploid population, and demonstrates a relationship between the critical mutation rate and the error threshold. The highest point at lower mutation rates in (b) appears to correspond to where the curve in (a) starts to ascend. Likewise, by the time the curve in (b) has descended to its lowest, the curve in (a) has reached its highest. This shows the transition of the population favouring peak 0 to favouring peak 1. The transition occurs around the critical mutation rate. At less than 50% loss of peak 0, individuals are still moving from peak 1 to peak 0. The critical mutation rate concerns the loss of individuals from peak 0 to peak 1, therefore the critical mutation rate should not be considered to be at a point where there is still a significant transition in the other direction (implying there is still a peak 0 advantage). In the top graph in Figure 6 (b), it can be seen that for smaller population sizes (less than 50), the curve does not fall below approximately 70% loss of peak 1. Considering the equivalent portion of the graph, Figure 6 (a) suggests that considering a peak loss of anything much less than 50% will be redundant when the population is small. The critical mutation rate should be considered not as a single value at the midpoint, but rather as *lying within a range of values with a lower limit of 50% loss of the high, narrow peak*.

These contributions provide the key insight that the critical mutation rate, at which individuals with greater robustness to mutation are favoured over individuals with greater fitness, has an exponential dependence on population size in both haploid and diploid populations, the latter in a system modelled on the biological process of meiosis. This is in contrast to previous studies which identified that critical mutation rate was independent of population size. Our results show the effect of population size to be particularly strong in small populations with 100 individuals or less. When a population’s size drops to this level, its critical mutation rate can be exceeded (in the absence of rapid mutation rate control) leading to loss of genetic material and a feedback spiral into further population size decline, genetic loss and on toward extinction. Population decline can lead to loss of fit genetic material that may be difficult to recover in very small populations. We have not identified a threshold for extinction, but have highlighted the fact that smaller populations experience error catastrophes, during which a population shifts in genotype space to areas of the landscape with lower fitness, at lower mutation rates. Such shifts indicate a population has become less well adapted to the current environment; smaller populations are at greater risk of extinction due to the presence of fewer individuals in the first place, with a smaller gene pool. Future work may determine the effect this has on population extinction and recovery, using parameter values within ranges found in nature. Testing the efficacy of different population management and conservation strategies (such as combining or mixing multiple small populations) on populations of varying sizes could also highlight the importance of considering population size and its relationship to genetic loss, as demonstrated here, during the decision making process.
